# Gays vs. Russia: media representations, vulnerable bodies and the construction of a (post)modern West

**DOI:** 10.1080/13825577.2017.1369271

**Published:** 2017-11-16

**Authors:** M. Katharina Wiedlack

**Affiliations:** ^a^ Department of English and American Studies, University of Vienna, Vienna, Austria

**Keywords:** Russia, Western hegemonies, LGBTIQ+, vulnerability, victimisation, modernity

## Abstract

This essay analyses the recent focus on Russian human rights violations in Anglophone media, scrutinising the ideological agenda of the visual politics which strategically foreground victimised bodies of Russian dissidents. Notwithstanding the importance of a critique on human rights violations, the article points to the unwanted but very real side effects the current mediatisations of violence have, from structural victimisation and the creation of ‘gay martyrs’ to the resignification of the West as progressive and ‘gay’ and Russia as backward and heterosexual. A close reading of popular press photographs of wounded Russian gay youth and the textual context – arguably representative for the Western media focus on the ‘Eastern’ violation of human rights between 2012 and 2014 – serves to illustrate how an image of Russian nation and Russian state politics is forged within Anglophone media discourses meant to reinforce the positive identity of the self-same by evoking pity, empathy and a responsible helpful attitude toward the endangered othered. The essay argues that Anglophone media’s focus on the vulnerability of Russian LGBTIQ+ bodies, consciously or unconsciously, reduces the subjects to this vulnerability, confirming feelings of moral superiority within the enlightened audience. The study highlights the important role that Russia’s vulnerable citizens play not only in the construction of values such as ‘tolerance’ and ‘acceptance’ and evaluations like ‘progressive’ and ‘modern’, but also in perceptions of the nation and its people and the reaffirmation of the dualistic divide between ‘The East’ and ‘The West’.

## Introduction

This article investigates the recent discourses on Russian LGBTIQ+^1^ issues in Anglophone media. It focuses especially on the visual politics of such discourses, which often present victimised bodies of Russian dissidents – beaten and frightened young female feminists, gays and seldom lesbians. It analyses representations of these bodies, and their semiotic significance in relation to the Russian nation and Russian state politics. Pointing to overrepresentations of Russian white gay cisgender male bodies within Anglophone media articles, concerning LGBTIQ+ issues in general, the article criticises such reductionist focus and attention. Moreover, it argues that by focusing only on white, gay, predominantly male and young bodies, the full extent of structural, social and political violence against sexual and gender minorities in Russia, as well as the recipients of this violence, becomes invisible. Media discourses in this vein do not only victimise the Russian gay men, but privilege them as primary recipients of Western compassion and solidarity. From a feminist standpoint, the neglect of lesbians, transgender and gender non-conformist, older bodies, bodies with disabilities or bodies of colour must be understood and criticised as the continuation of a male misogynist, ageist and transphobic gaze through Anglophone media. This does not mean that the journalists and photographers whose works will be discussed are necessarily misogynists, ageists or transphobes. However, their work can become (mis)appropriated by editors and news agencies who arrange them in a problematic way that supports North/Western hegemony rather than Russian LGBTIQ+ agency.

Media discourses on human rights and LGBTIQ+ issues in Russia exploded when the so-called ‘anti-homosexual propaganda law’^2^ was introduced in summer 2013, and such interest continued throughout the Sochi Winter Olympics in February 2014. Most recently, discussions flamed up again owing to the unsettling reports of anti-gay violence in the region of Chechnya in April 2017.

In this article, I question the cultural and ideological implications of the representation of young, dissident, gay bodies within liberal Anglophone media to disclose how subjects are reduced to their essentialised vulnerability for the sake of setting a moral exemplar. I analyse the connection between vulnerability and formations of values, such as ‘tolerance’ and ‘acceptance’, and the concepts of modernity and progress. I question the place of Russian citizens’ vulnerable bodies vis-à-vis the Russian state, and by association the Orthodox Church^3^, and mainstream society in contemporary cultural discourse as seen in news magazines’ photo reports or photo documentaries, social media and daily news. I will show that representative Anglophone media discourses locate gays in the centre of ‘Russia’s Culture Wars’ (Trudolyubov, 2014) – the negotiation of cultural values through the rejection of liberal socio-political ideas and concepts. Arguably, the media focus on Russian gays’ vulnerability in the face of physical and psychological attacks portrays Russia unfavourably as brutal, backward and anti-modern. Moreover, the media construct Russia as distinctly and decidedly different from the (European) North/West, which in turn appears united by its shared values of tolerance and appreciated diversity – reduced to the social and political inclusion of some (white) lesbians and gays.

I analyse the widely circulated press photographs of two young gay victims of homophobic violence – Kirill Fedorov and Dmitry Chizhevsky, as two of the many examples of representations featuring on corporal media pages, and frequently reposted on personal and private social media newsfeeds on Facebook or its Russian equivalent, Vkontakty. I discuss the pictorial representations by relying on the methodology of a ‘critical discourse analysis’ (Wodak, 1999), based on works of the heritage of Orientalism, North/Western Enlightenment and modernity (Neumann, 1999; Puar, 2007, 2013; Wolff, 1994), coupled with theoretical works reflecting on the post-socialist and post-Soviet context (Kulpa and Mizielińska, 2011, 2012). With Clifford, I contend that discourse analysis

is always in a sense, unfair to authors [here, especially the photographers]. It is interested not in what they have to say or feel as subjects, but is concerned merely with statements as related to other statements in a field.

(Clifford, 1988: 270)

In this vein, I draw on the insights of theories on embodiment and vulnerability (Butler, 2006; Fineman, 2008; Kaul, 2013; Razack, 1998) to decipher the cultural meanings attributed to the representations at play and tackle their functions in the intermedial discourses on Russia and human rights. Discourse analysis looks at the represented bodies and their meanings at the intersection of gender, sexuality, race, class and age in their full complexity. I focus on how ‘“acceptance” and “tolerance” for gay and lesbian subjects have become a barometer by which the right to and capacity for national sovereignty is evaluated’ (Puar, 2007: 336) within current media reports. Robert Kulpa and Joanna Mizielińska identify a problematic concept of time and progress within hegemonic discourses on queerness, which confirms the North/Western model as forward-thinking and the Eastern context as lagging behind, or a ‘lesser’ copy of the North/West. I combine their theories with theories of vulnerability to arrive at a better understanding of the semiotics of vulnerable bodies, especially of Russian white male victimised bodies. In doing so, I aim to trace the instrumentality of the construction and display of vulnerability in the invention of geographical, social and cultural East/West differences and similarities. The article relates theoretisations of geotemporal paradigms in the post-socialist and post-Soviet context (Kulpa and Mizielińska, 2012) to postcolonial thinking (Puar, 2007; Ferguson and Hong, 2011) to illuminate intermedial meaning production in a new way.

## The open wound: Dmitry Chizhevsky and Kirill Fedorov, vulnerability and ethics

Dmitry Chizhevsky was the victim of a homophobic attack at an LGBTIQ+ community gathering at the LaSky HIV charity centre in St Petersburg on 3 November 2013 (Morgan, 2013). Chizhevsky was wounded by a rubber bullet and lost his eyesight. His pictures – especially pictures taken after the attack, showing his wounded eye covered with white bandages – became internationally distributed pieces of ‘evidence’ against contemporary Russia. The now defunct *St. Petersburg Times* featured a page-sized picture of Chizhevsky sitting with one eye covered with a gauze bandage in a hospital bed (Queer Nation NY, 2013); the activist group Queer Nation NY re-posted the article and picture with the caption statement ‘This is why we fight’ on their homepage in November 2013, supporting the call for a boycott of Russian vodka in solidarity with Russian LGBTIQ+s as well as Queer Nation NY’s street protest with regards to the then upcoming Sochi Winter Olympics in February 2014. Simultaneously to the *St. Petersburg Times* article, the online news magazine *LGBTQ Nation* (Staff Reports, 2013) published a picture and text on 4 November 2013, followed by the news blog *The Russian Reader* (2013) only a couple of days later. These, as well as other reports in newspapers (*St. Petersburg Times* in Queer Nation NY, 2013), magazines (Nissen, 2014), activists’ web pages (Global Forum on MSM & HIV, 2015; Queer Nation NY, 2013) and other community media (#LGBT on Twitter and Facebook), presented Chizhevsky as one victim of a group of LGBTIQ+ people who are now the targets of ‘anti-gay hysteria in Russia’, created or at least supported by the then recent introduction of the so-called ‘anti-gay propaganda laws’.

One picture I will discuss as representative for all the reports is Misha Friedman’s shot of Chizhevsky. It conveys the sentiment that all of the pictures have in common. Moreover, it is arguably the most widely distributed photo of Chizhevsky, owing to Friedman’s collaboration with the Pulitzer Center in New York.[Fig F0001]

**Figure 1. F0001:**
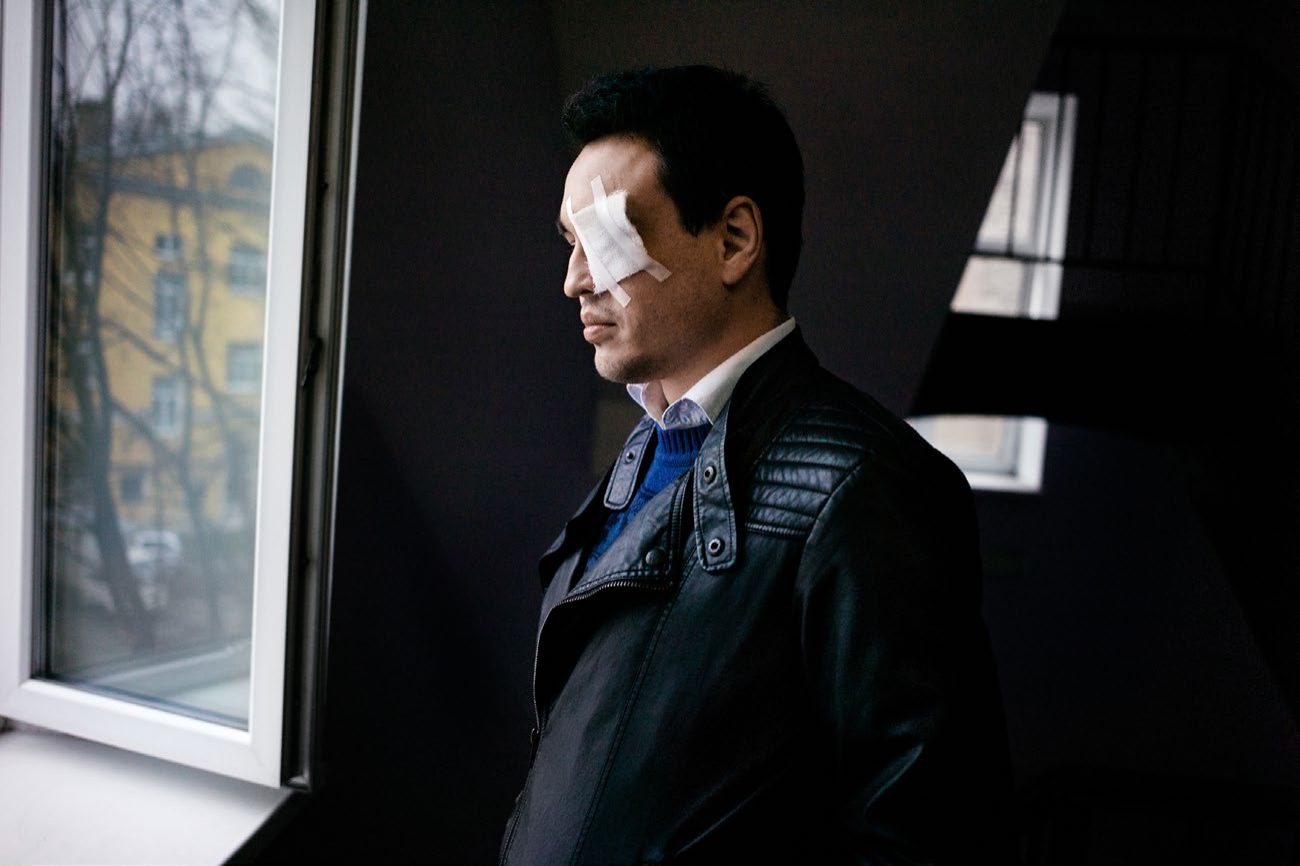
Dmitry Chizhevsky after being shot with a rubber bullet on November 3, in an apartment in Saint Petersburg. Reproduced with permission by Misha Friedman.

Friedman’s picture focuses intensely on Chizhevsky’s face, although we can only barely make out its melancholic expression. The darkness of the room and Chizhevsky’s black leather jacket make the cotton bandage over his eye appear very bright, immediately attracting the gaze of the viewer. Like his face, the view outside the window is not directly revealed; it is only mirrored through the open window’s glass. Although we cannot see Chizhevsky’s gaze, we know that he is looking outside, into St Petersburg’s autumnal moodiness. The colours of the photo enhance the melancholia – black, white, grey, blue and just a little bit of the pale pink facial skin and a yellow house outside. The picture draws on art historical conventions as it centres the upright young man. It is not merely a documentation, it is a piece of political art, aesthetic not by accident, but by the arrangement of an artist. It is ‘aesthetic’ in the most archaic sense of the term, as it is a ‘discourse of the body’ (Eagleton qtd in Buck-Morss, 1992: 6). ‘It is a form of cognition, achieved through taste, touch, hearing, seeing, smell – the whole corporeal sensorium’ (6). But it is also aesthetic in a more enlightened, modern, Kantian sense, evoking a value judgement, as it reveals the complicated relationship between the representation of ‘truth’, values, authorship and the form of art. It is most of all post-modern, as it poses the question not just of the aestheticisation of ‘any truth’, but of violence, pain and suffering. Yet, this question, pressing to the researcher, can be ignored or rather not raised by an audience whose affects, empathy and politics sympathise with the young gay man. Ignoring the artificiality of this piece of art, product of processes of arrangement and cropping, the audience is tempted to understand the piece as revealing a truth or reality. The text of the article, as I will show below, supports such a reading.

Chizhevsky’s half-illuminated figure is set against the greyness of the outside world. The monotone colours of the exterior suggest a coldness and harshness, which positions Chizhevsky’s physique as vulnerable. The vulnerability of LGBTIQ+s is thus represented through the wounded body of Chizhevsky, although we can only assume the wound covered by the white bandage. What furthers the impression of vulnerability is that we cannot meet Chizhevsky’s gaze, as the bandage itself deprives the photographic subject from the very act of looking, and visibility alike.

Vision signifies individuality, a subject position, it is the ‘window on the world’, as well as the ‘mirror of the soul’. In his extensive French cultural history on vision, Martin Jay (1993) points out that ‘[t]he eye … is more than the passive receptor of light and colour. It is also the most expressive of the sense organs, with the only competitor being touch’ (9). The eye signifies agency, because it ‘can obey the conscious will of the viewer’ (10) through its variable modes, ‘[r]anging from the casual glance to the fixed glare’ (10). Furthermore, perception and ‘language as a generic phenomenon’, and difference are inextricably connected, as ‘the universality of visual experience cannot be automatically assumed, if that experience is in part mediated linguistically’ (9). What does it mean in this light, that Chizhevsky cannot see and we cannot meet his eye? Chizhevsky’s inability to face our gaze as spectators deprives him of any ocular agency, but also makes him metaphorically mute, speechless, without language. The Enlightenment’s role in the ‘privileging of sight so often taken to characterise the modern era in general’ (97) needs to be considered in relation to Chizhevsky’s visually ‘mute’ figure that is so central in debates about the ‘barbarism’, backwardness and incivility of Russian human rights violations. Furthermore, we need to take into account that the picture illustrates a journalistic article about the very same Russian context, speaking for the Russian victim. The inability to speak himself through his eyes, and the pose of facing the window, rather than the spectator, emphasise the danger of the Russian environment Chizhevsky is only half able to face now, with only one functioning eye, and it releases the spectator from all responsibility for the process of looking, observing or gazing. To put it differently, the fact that our gaze is informed through (North/Western) cultural ideas, images and biases seems irrelevant considering that this grey St Petersburg outside is not a safe place for this gay man in particular, and LGBTIQ+s in general.

The dark room where Chizhevsky is located emphasises this perspective, as it is a metaphor for the living conditions for LGBTIQ+s in Russia: it is a claustrophobic space, a closet and every outing, here signified through the cold outside world, bears violence and harm. The positioning of windows, however, is extremely interesting and might offer the possibility for a counter-reading or an irritation of the benevolent victimising gaze. A closed window in the background is contrasted with an open one through which he is contemplating the outside reality endangering him. The open window matches his open wound. As spectators we cannot see what he sees, just as we cannot see his wound beneath the bandage. This arrangement can be read as signifying the difficulty of empathy, sympathy, of tolerance, of feeling, voicing or picturing the pain of other(ed)s. Moreover, Chizhevsky’s inability to meet our gaze, his inability to look back at us, could teach us about the aims of our solidarity – of supporting the agency of Russian LGBTIQ+s, rather than saving or rescuing them. However, the text accompanying the pictures sabotages such a reading by presenting the West as single adequate environment for LGBTIQ+ lives.

The first article that, to my knowledge, featured Chizhevsky’s picture taken by Friedman further emphasised its exemplification of the vulnerability of LGBTIQ+s in Russia. Nora Fitzgerald and Vladimir Ruvinsky, in their *Politico* article ‘The Fear of Being Gay in Russia’ (2015), portray Chizhevsky as ‘just one of a growing number in Russia’s LGBT [*sic*] community who’ve been attacked or harassed in what has become an unprecedented crackdown’. Furthermore, they contrast Russia with ‘most of the West, [where] gay rights has [*sic*] seen startling breakthroughs in the last decade’, only to conclude that ‘Russia has not just been left behind, but has become demonstrably worse and more dangerous’ in this comparison.

Fitzgerald and Ruvinsky present Chizhevsky and LGBTIQ+s as ultimate victims of violence. They understand Russian LGBTIQ+s as a ‘community that was just beginning to organise [and] found itself under assault, the target of a deep-seated Russian homophobia that had now been embedded in law’ (Fitzgerald and Ruvinsky, 2015). Their focus on LGBTIQ+s allows them to activate a development paradigm that understands the North/West as a positive model of acceptance and diversity, toward which Russia had previously moved, until the Russian state changed its course towards authoritarianism and monoculture. Friedman’s photograph transports this meaning of backwardness onto the visual level through the darkness that surrounds Chizhevsky’s illuminated face and the bandage. Chizhevsky epitomises the vulnerability of LGBTIQ+s’ bodies violated by the homophobic Russian society and the Russian state, a vulnerability signified not only through his upright but thin figure and downward-looking gaze, but through the hidden wound behind the bandage.

Misha Friedman and journalist Nora Fitzgerald, who were both grantees of the Pulitzer Center and supported by the Arcus Foundation, made several public appearances where they told Chizhevsky’s story. All of these events – for example at the Religious Freedom Center of the Newseum Institute, where Chizhevsky himself spoke as well, and at a LGBTIQ+ conference at Harvard – were advertised with Friedman’s picture of Chizhevsky. Moreover, the image was shown at the photography exhibit in the Monroe C. Gutman Library Gallery in Cambridge as well as in the Photoville 2015 Container Exhibition (10–20 September 2015) at the Brooklyn Bridge Park, as part of Friedman’s series ‘The Iron Closet’. It was also featured in a *CBS News* report of the entire Photoville 2015 Exhibition that included more than 48 cargo containers, each designated to either a topic or an individual artist. The text announcing ‘The Iron Closet’ series read ‘Being gay in Russia is lonely and extremely dangerous. Being different is not celebrated. It is prosecuted’^4^.

The Norwegian Mads Nissen captured an image of Chizhevsky that became almost as equally well known as Friedman’s. It was published as part of a *Newsweek* photo essay on ‘the dangers of being gay in Russia’ (Nissen, 2014). The photograph from Nissen’s series that reached iconic status within North/Western news and social media, however, is not the image of Chizhevsky’s wounded face, but a picture of the St Petersburg-based activist Kirill Fedorov[Fig F0002].

**Figure 2. F0002:**
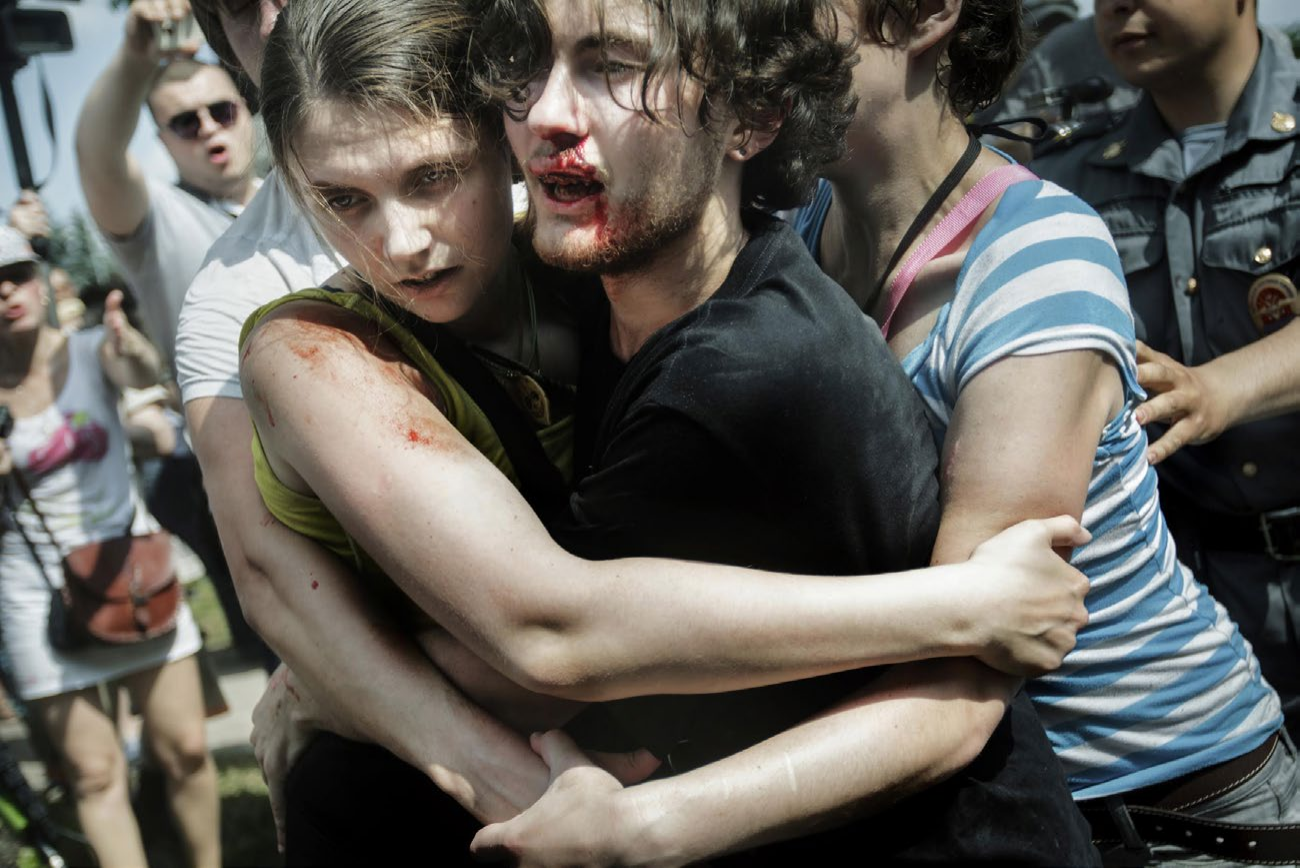
Kirill Fedorov attending a pride rally in Saint Petersburg in Summer 2013, after being beaten by national-conservative extremists. Reprinted with permission by Matts Nissen.

The photograph shows the bleeding LGBTIQ+ activist Kirill Fedorov at a Pride rally in St Petersburg in summer 2013. The arrangement of Fedorov’s body, his bleeding wound, pale face, three-day beard and long brown hair, surrounded by two women figures, reminds one of a Christian martyr. The two women are reminiscent of the two Marys in the Christian iconographic tradition depicting the Passion of Christ. Significantly, the text explaining the picture does not reveal their names. They are mentioned as Kirill Fedorov’s friends. This little detail is very significant in terms of an analysis of the gender aspects of North/Western LGBTIQ+ solidarity projects, which rarely ever focus on women and lesbians.

The Russian artist The Blue Iconostasis (2015) has produced an oil painting of the picture in the style of a traditional Russian Orthodox iconography. What makes the picture a gay or queer icon is not just its arrangement of wounded bodies, but its ‘glorification’ within Anglophone and international media. One fan even produced a video as a tribute to Mads Nissen’s photo essay that begins the slide show with Fedorov’s iconic picture paired with the great pathos of Wolfgang Amadeus Mozart’s and Herbert von Karajan’s music (Davenne, 2016). Mainstream news equally celebrated Nissen by presenting the photograph of Fedorov, including the Weekly Photography corner at the *Culture Magazine* at i24news channel (Gurovich, 2015). The occasion for a report on Nissen’s work was his winning the World Press Photo of the Year award in 2014.

Founding figure of feminist interventions in intermediality, Mieke Bal understands ‘iconography as a useful yet limited code [that] contains and generates verbality in the very core of art history’ (1991: 178). She describes iconography as a way of ‘reading’ of a detail in a picture. Her reading connects the ‘signs with the absent item they stand for’ (215). I argue that the detail that we are turned to through the iconographic arrangement in the photograph is Kirill’s wound. But what does the iconographic representation of Kirill stand for? What is the ‘absent item’? The sacrifice for being ‘true’ to oneself, the very mechanism North/Western gay identity is based on? Or the violence against him, which we learn is sanctioned by the Russian state itself? In any case, Fedorov as a martyr evokes affective responses of pity and shame, the willingness to help, resulting in his spiritual elevation. Such feelings are normative as they are sentimental.

Such worshipping of a queer icon or martyr is not uncommon within Anglophone contexts. Patricia Juliana Smith (1999: XV) argues that ‘[t]he worship of idols – of “false” gods – is [indeed] an integral part of queer culture, particularly in times when homosexuality is most severely proscribed’. Dominic Janes equally argues that the Catholic Christian imagery of the martyr played a key role in the evolution of the culture and visual expression of homosexuality and male same-sex desire in the nineteenth and twentieth centuries and continues to influence queer culture today. Janes traces the beginnings of what he calls ‘queer martyrdom’ back to the nineteenth-century Church of England. He considers how queer martyrdom provided inspiration for artists looking to communicate their own feelings of sexual deviance during Victorian times and beyond. With reference to the work of Oscar Wilde and Derek Jarman^5^ Janes argues for the persisting significance of the queer martyr as an inspiration for expressions of homoerotic desire. ‘The life and work of Derek Jarman’, Janes writes (2015: 29), give ‘testimony to some of the ways in which the older model of queer martyrdom recovered relevance and importance in the 1980s’. Janes emphasises the ‘use of melodrama, posing, and stylisation as a way of engaging an audience’ (27) within the history of queer culture and reads it in close proximity to the stylisation of the martyr in Christian tradition. While he speaks to the context of the UK, scholars such as Smith read the celebration of popular figures by queer audiences during the 1960s as creation of ‘martyrs/icons’ (Smith, 1999: XV) in the US-American context. Figures such as Andy Warhol, for example, were celebrated for their pain and suffering, deputising for all suppressed homosexuals (XV).

Janes (2015: 5) offers a possible explanation for the appeal of martyrs within queer cultures. The ‘visual images and imaginary visions of suffering’ inherited from ‘ecclesiastical contexts’ were used to ‘develop concepts of male same-sex desire that projected the self as dutiful and penitent rather than shameful’ (5), Janes argues. Anglo-Catholic images of martyrs ‘could, subsequently, come to be used by others as a way of reflecting on and contributing to the construction of their own notions of queer sensibility’ (5). The depictions of queer martyrs became interpreted as ‘queer triumph over adversity, or sad tableaux of sexual failure’ (5).

The photograph of the bleeding LGBTIQ+ activist Fedorov conveys both of these qualities in the eye of the Anglophone press. It is a celebration of queer resistance and integrity as well as a sad image of sexual suppression. ‘Martyrdom is a social formation and requires the witness not just of the martyr but of others who will attest to one’ (9). It is a condition ‘that can be applied to create a sense of exalted drama around the sufferings and privations of sexual and gender deviants’ (12). It can be employed by an individual her/him/itself, but it can also be ‘imputed to others’ (12).

What makes the martyr a martyr, however, is not just an audience that understands his/her/its precarity and vulnerability, but that has also a clear vision of his/her/its oppressor as a hegemonic power. In Nissen’s picture, the martyr Fedorov is set against the background of a yelling and gesticulating aggressive crowd of Russian people. The arrangement of the bodies constructs the vulnerable gay body as much as it establishes its counterpart – the violent Russian bodies of the homophobes. The text that accompanies the picture in different news media further emphasises this formation. North/Western media has argued that Fedorov’s photo shows ‘The Brutal Violence Inflicted on LGBT People in Russia’ (Buzzfeed, 2015; Newsflow24, 2015), ‘portraying how extremely difficult it has become living life as a Gay, Lesbian, Transgender or Bisexual at Russia nowadays’ (Gurovich, 2015). It has appeared in numerous tumblr (Kachkinlovesu, 2015) and Twitter feeds, for example as part of the Twitter feeds #LGBT #NOH8Worldwide #StopHomophobia campaigning for the film ‘The World According to Homophobes’, which focuses on international homophobia.^6^ The teaser of the film shows people speaking out against homosexual acts, and some gay bashings on streets; all the overwhelming amount of street violence cut from news out of Russia. In sum, all of these news portrayals leave the impression that Russian homosexuals are powerless victims, helplessly facing their oppression by all-encompassing Russian homophobia.

## Looking at the vulnerable – feeling injustice

Fedorov and Chizhevsky are presented as vulnerable in the most literal sense: the English word ‘vulnerable’ comes from late Latin ‘vulnerabilis’, which translates into ‘wounding’ and ‘vulnus’, which means ‘wound’ (Harper, 2016). The visibility of their violation further makes the pictures speak, connects them to the text, and brings the two men’s white bodies into our consciousness as bodies we can relate to, we can be in solidarity with. The pictures show the faces as sites of physical wounds, but also remind us that the face is what makes us recognisable as individuals, as well as humans among humans, emphasising what we have in common. Like the face, our individual corporeal vulnerability defines us universally and ontologically as human beings (Butler, 2006; Ricoeur, 2007), but the moment vulnerability appears, it signifies individuality. The visibility of specific individual vulnerability signifies the person’s or group’s ‘susceptibility to harmful wrongs, exploitation, or threats to one’s interests on autonomy’ (Rogers, Mackenzie and Dodds, 2012: 3). Most importantly, the visibility or appearance of vulnerability signifies social hierarchies. In this logic ‘the especially vulnerable are those who, due to inequalities of power, dependency, capacity, or need, are less able than others to protect themselves’ (3).

Feminist and disability scholars have turned to vulnerability to theorise the relationship between individuals, society and nation states. Judith Butler (2006: 27) invokes vulnerability as a universal human experience and point of interconnectedness to argue for solidarity, social and state responsibility. While Butler focuses on shared vulnerability as human condition, Kate Kaul (2013), Sherene Razack (1998) and Martha Fineman (2008) scrutinise the particularity of individual vulnerability as to what differentiates subjects from each other. They draw attention to the fact that vulnerability is a central category justifying the need for state protection. Human rights lobbying groups such as UNESCO’s International Bioethics Committee equally focus on vulnerability, arguing that its recognition may build a ‘bridge to greater solidarity, and that a commitment to respect for vulnerability is a necessary constituent of the political responsibility of states’ (Rogers, Mackenzie and Dodds, 2012: 8).

However, vulnerability also implies dependency. And although it is crucial for a state to identify the different needs of different people, the identification of certain groups and individuals as vulnerable is not unproblematic. The structural connection of vulnerability and dependency, for the liberal subject, inevitably refers to the status of children (Fineman, 2008: 2; Kaul, 2013: 102), hence, not (yet) full citizen rights and abilities. Furthermore, vulnerability and dependency create feelings of ‘pity’. The ‘ableist gaze’ (Razack, 1998: 132) reduces persons with a visible vulnerability to ‘icons of pity’ (132), as Razack has pointed out. Although she uses the term in reference to disabled women, I argue that Fedorov and Chizhevsky are equally produced as icons of pity through the ableist and heteronormative North/Western gaze of Anglophone media.

It is indeed pity that is the emotional response to their vulnerability and the wish to save them and others like them. Pity, although it might be connected to admiration, is not respect; it is rather an emotional state in which the person who feels pity complies in oppressing others by feeling superior, powerful and able. Proliferating the pictures of the wounded faces of Fedorov and Chizhevsky, public media sacrifices them again and again to illustrate the nation state’s injustice and public violence towards sexual minorities in Russia.

Simultaneous with the philosophical reframing of vulnerability as a chance to rethink and reimagine human connectedness, media discourses insist on vulnerability as a problem to be solved through protection of the state or international (meaning US) powers. The pictures of Fedorov and Chizhevsky show vulnerability to make us feel that the state is responsible and, as citizens of states that are ‘good’, morally self-conscientious states, nations or supra-national powers, we feel the responsibility. At the same time, we detach the vulnerable subject from any agency; we do not ask what the subject wants, because we already know what is best for her, him, them.^7^ Media produces vulnerable bodies to point out the injustice of oppressive forces endangering these bodies. Yet the specific reason for the bodies’ vulnerability, the concrete source of violence and its political nexus, is no longer presented. Labelled as gay, their status as sacred figureheads of modernity and modern values is established and it is no longer relevant what the political issues had been before – that the activist Fedorov was beaten by right-wing activists – nor is it important in which specific context Chizhevsky was shot. The reason these subjects are vulnerable, according to the media, is rather the wrongness and backwardness of the Russian state, because ‘[u]nder President Vladimir Putin, Russia has been sliding back toward the Middle Ages’ (Nemtsova, 2015).

## Localising Russia – the geography of Russian backwardness, North/Western modernity and homonationalism

Fedorov and Chizhevsky become central figures within the New Cold War between Russia and ‘the West’, not as individuals, but as representatives for the victims of Russian authoritarianism and homophobia. This New Cold War (Commission on Security and Cooperation in Europe, 2011; Sakwa, 2008)^8^ neither started nor evolved around the topic of LGBTIQ+ rights. However, media reports on LGBTIQ+ issues were part of these New Cold War discourses that created notions of Russia as authoritarian, unfree and undemocratic re-entered popular media as well as government commentary. Such discourses favourably position North/Western Europe and the US as counter-model. Such narratives use metaphors and imageries of past decades (Neumann, 1999; Wolff, 1994), creating meanings of North/Western progress and Russian stagnation or backwardness. Larry Wolff (1994) and Iver Neumann (1999) argue that the foundation of the contemporary cultural signification of Russia was established not within the Cold War, but actually with the Enlightenment’s invention of Eastern Europe as a cultural and intellectual construction with the goal to create a stable concept of North/Western superiority and development. Within this framework, philosophers stigmatised Eastern Europe, including Russia, as a paradoxical locus of difference and similarity, in between North/Western European civilisation and the ‘barbarian Orient’. Today ‘[m]any area specialists [equally speculate] about the prospects for a convergence of the “uncivilised” Russia with a united Europe’ (Dutkiewicz, 2011: 10; Kagan, 2004). The framework within which the evaluation takes place is modernity. Reports about Crimea draw on the same sentiment with headlines such as ‘Caught in the grip of the Russian bear’ (Pollard, 2014), pointing out that ‘Russia is moving backwards thanks to Putin’ (The Hamilton Spectator, 2015). The opinion that Russia is backward and anti-modern is part of many discourses – not restricted to LGBTIQ+ issues. However, a focus on LGBTIQ+ rights comes up especially frequently in references to modernity. Particularly liberal media such as the *Huffington Post* frame the political struggles in Russia as questions of ‘Modernity vs. Forces of Yesteryear’ (Simonyi, 2015). Moreover, LGBTIQ+s are increasingly conceptualised not only as vulnerable towards Russian violence, but as carriers of culture and sophistication. Given the opportunity through their country’s development, which means ‘[l]iberated from the pressures of discrimination, [the LGBTIQ+ community] will be able to exercise their creative power to the maximum’ (Simonyi, 2015).

In all these reports the Enlightenment’s notions of Russia (Baer, 2002; Stella, 2015) prevail, yet the very definitions of modernity and Enlightenment have altered. The queer and postcolonial scholar best known for identifying the transformation of contemporary ideas of Enlightenment, modernity, progress and civilisation within the North/West, especially the US-American context, is Jasbir Puar (2007, 2013). Puar argues that the changes in the relationship between sexuality, the state and capitalism within the global North/West have transformed or actualised the concepts of civilisation, progress and modernity. The dominant narrative of progress, used to positively signify a state, nation or region and its population, has changed insofar as it now includes an aspiration to gay rights. This change of narrative, however, is ‘built on the back of racialised others, for whom such progress was once achieved, but is now backsliding or has yet to arrive’ (Puar, 2007: 337). In other words, the positive progressive attitude is constructed in opposition to racialised populations, who allegedly threaten its continuation. The racialised subjects become signified through their heritage, their racialisation and their presumed ‘different’ values. Puar developed her concept to understand how US xenophobia and Islamophobia structure racialised Muslim subjects to delegitimise their cultural and territorial environment, but the mechanisms of othering and delegitimisation through an identification and rejection of homophobia in ‘the other’ is not dependent on or restricted to Islamophobia or Muslim subjects.

Russian Studies scholars Brian Baer (2002) and Francesca Stella (2015) equally identify the discursive field of (homo)sexuality as an ideological site where ideas of North/Western progress and values are propagated/praised and Russian othering takes place. They criticise previous works on Russian sexualities as contemporary reiterations of the Enlightenment’s development paradigm. Such representations situate Russia ‘on the periphery of Western Europe’ (Baer, 2002: 502). In contrast with the peripheral Russia, North/Western Europe appears to be modern because of its seemingly ‘egalitarian sexuality (the global gay)’ (502). The negotiation of cultural values, however, is neither played out within the sphere of the legal or theoretical debates, nor on the level of anonymous populations, Russian majorities and minorities; rather, it takes place in the midst of the cultural war, where real as well as fictional figures emerge, marked by their victimisation through Russian forces, President Putin and his ‘minions’ (Myers, 2014).

Anglophone media frequently compares Russia to North/Western nations to signify the developmental direction it is taking. ‘As the lesbian and gay minorities of France, England and Wales celebrate the recent laws permitting same-sex couples to marry’, one reporter writes for example, ‘Russia is marching briskly in the opposite direction’ (Wintemute, 2013). Michelle Rivkin-Fish and Cassandra Hartblay (2014: 98) emphasise that North/Western discourses frame ‘Russia’s anti-gay legislation as evidence of Russian authoritarianism’ and ignore ‘the collaborations between U.S. Evangelicals and Russian conservatives’ that supported the law, in favour of portraying ‘Putin as a rogue despot, exceptional among contemporary political leaders’. Instead of an evil act of the overly powerful Putin, they understand ‘Russia’s gay politics as yet another example of global cultural politics between religious fundamentalism and secular morality that plays out every day in the West’ (98).

Robert Kulpa and Joanna Mizielińska show in their elaborations on the geotemporal paradigm of Central and Eastern Europe, that the invocation of queers often produces a problematic concept of time and progress in which the North/Western model can only ever be seen as forward while the Eastern counterpart can only ever appear far behind and in need of catching up. The East appears as a ‘poor cousin’ to the West, which ‘is now, supposedly, catching up with normality (a.k.a. the “West”)’ (Kulpa and Mizielińska, 2012: 23). Seeing Fedorov’s and Chizhevsky’s victimised bodies, and reading the texts about gay rights in Russia, bring our already existing knowledge about the lagging East to the fore. Moreover, we read and understand the bodies as subjects of comparison, between the North/West and the East, in the struggle for modernity.

Jasbir Puar’s conceptual framework of ‘homonationalism’ can help us to understand why the press turns to ‘acceptance’ and ‘tolerance’ for gay and lesbian subjects to evaluate ‘the right to and capacity for national sovereignty’ (2007: 4). Puar understands homonationalism as ‘a facet of modernity and a historical shift marked by the entrance of (some) homosexual bodies as worthy of protection by nation-states’ (2013: 337). It is implemented by the US and other North/Western states^9^; however, popularised across the globe by media. Within homonationalism, ‘western-style gay liberation’ (Stella, 2015: 138) as well as gender equality ‘represent … the high point of modernity’ (Binnie, 2004: 85), and gays and lesbians are, accordingly, created as visible signs of modernity and progress. Helen Lenskyj (2014) and Fred LeBlanc (2013: 7) identify the ways in which public discourse in North America and the UK idealise ‘the West … as progressive and liberal against a foreign culture, in this case Russia, despite the inability for some Western states to extend full rights to LGBTIQ+ subjects’ with Puar’s term as homonationalistic. In close readings of US and UK national debates in the wake of the 2014 Sochi Winter Olympics, LeBlanc (7) argues that LGBTIQ+ activists and public figures presented the US and UK as ‘gay-friendly, tolerant, and sexually liberated society’ and enacted ‘pro-national, pro-Western, and anti-Othering scripts that continually (re)produce the [Russian] Other as intolerant, sexually repressed, and uncivilised’. He goes so far to say that ‘in 2013 Russian homophobia seems to have momentarily trumped Arab homophobia in the media’s discussion’ (7).

Understanding homonationalism as an actualisation of Newmann’s and Wolff’s enlightenment paradigm, where Russia is positioned in-between the enlightened and civilised North/West and the backward and racialised Orient, we can understand how current discourse cements Russian bodies’ signification as in transition, leaning towards backwardness, while condemning the Russian nations as authoritarian and anti-modern. Although Russian citizens are increasingly understood as conservative and backward, accusations of homophobia are mostly reserved for the state and its authorities. LeBlanc and Lenskyj focus exclusively on text-based discourses. Puar’s concept, however, speaks to the interrelations of different forms of media in localising not only nations, but actual bodies within the developmental paradigm towards modernity. On the intersection of the visual wound, the textual information about human rights abuses and the already existing knowledge about Russia’s civilisation status emerge the feelings of belonging to the West, to a Western nation, but also of solidarity and pity, and a commitment to progress, tolerance and human rights.

## Conclusion

The Anglophone media produces discourses on Russian LGBTIQ+ issues, human rights and equality that locate Russia on the fringe of modernity at best and in the dark sphere of the anti-modern at worst. Newspapers, magazines, journals and websites offer images of beaten LGBTIQ+ activists, like the two examples of Fedorov and Chizhevsky discussed here, as visual evidence of Russia’s brutality and inhumanity. Liberal media choose the images of wounded bodies such as gay activist Fedorov and gay community member Chizhevsky, because such pictures can become attached to issues and values that are central to contemporary liberal rights discourses within the North/West. It was easy to present the two young gay men Fedorov and Chizhevsky as sacred figures and as martyrs. They appear to be painfully fragile, threatened by the overtly powerful Russian state and other violent agitators within the Russian population. In their frequency and wide dissemination, these pictures create an unbalanced focus on gay white young male bodies. It reduces LGBTIQ+ issues and questions of non-normativity to male homosexuality, problems of visibility and public life, and focuses almost exclusively on public physical violence. My intermedial analysis aimed to show how visual, verbal and affective representations of Russian gay men as queer martyrs creates a sense of physical, emotional and political vulnerability. Because such pictures suggest passivity and endurance, rather than action and agency, culminating in the notions of martyrdom and queer iconography, they lead to an exploitation of vulnerability and enhance the signification of Russian LGBTIQ+s as vulnerable only. Such aggravation of notions of vulnerability can easily get used to demonise Russia, and, as the many examples of news media representations show, it has been used like this. The focus on wounded sacred, mostly white, gay male and young bodies such as Fedorov and Chizhevsky, reduces the issues at hand to gay men and renders lesbian, trans*, other non-normative subjects as well as LGBTIQ+ political agents invisible. In other words, it ignores lesbians, transgender and other sexually and gender-nonconformist people, their challenges, but also their forms of resistance. Moreover, it supports a hegemonic notion of North/Western superiority, where the North/West can reaffirm its own post-homophobic society and attitudes. Thus, such a focus on vulnerability allows for a reduction of the victims of homophobic violence to mere objects of North/Western pity, in need of the help of North/Western actors. It creates a discourse on values and ethics that locates Russia on the move away from modernity – a modernity which, in turn, is equated with the North/West. This unifies Russia as one homophobic nation and culture, creating a notion of Russian dissidence as completely anti-Russian.

Most importantly, such discourses miss the problem at hand, which, arguably, is not the majoritarian homophobia of Russian people, culture or the state, but the propaganda of heterosexuality, procreation and the family as a Russian nation-building project (see also Baer, 2013: 51) that uses homophobia as expedient. Such a nationalistic heteronormativity that focuses primarily on the promotion of family and family values, however, is also very present within US and other North/Western contexts. The recent voicing of a homophobic and transphobic nationalism is similar to Russia’s patriotic, heteronormative and family-centred nationalism that uses homosexuality as its binary, unnatural and threatening opposition. Addressing Russian nationalism, accordingly, is not suited to positioning North/Western nations as favourably as the focus on homophobic violence and gay vulnerability does. However, addressing this mechanism could be a chance to go beyond the East–North/West binary that positions the North/West as model. It could allow us to look for ways to fight homophobia in more appropriate, less hegemonic ways, seeing Russia not as lagging behind, but regarding its nationalistic homophobia as a global trend with nationally different varieties and effects.

## Notes on contributor

***M. Katharina Wiedlack*** is Hertha Firnberg post-doc fellow at the Department of English and American Studies, University of Vienna. She has conducted research at New York University, UC Berkeley and Johns Hopkins University among others. She has taught Gender, German and Disability Studies at Lomonosov University Moscow, State University St Petersburg, State Technical University Novosibirsk, Charles University Prague and the University of Vienna. Currently, she is working on a research project on the construction of Russia’s most vulnerable citizens within US media, funded by the Austrian Science Fund. Additionally, she works for/with the research, art and culture network (https://bodypolitix.me) that focuses on queer post-Soviet pasts and presents as well as queer migration. E-mail: katharina.wiedlack@univie.ac.at

## Disclosure statement

No potential conflict of interest was reported by the author.

## Funding

This work was supported by the Austrian Science Fund [grant number T767].

## Notes

1. LGBTIQ+ stands for lesbian, gay, bisexual, transgender, intersex, queer, as well as sexually and gender fluid people. The reason for choosing the abbreviations instead of the umbrella term ‘queer’ is that in Russia the term ‘queer’ is used only by a small and elitist minority, whereas the terms ‘gay’, ‘lesbian’ and ‘transgender’ as well as other local terms are used quite frequently (see Wiedlack and Neufeld, 2016: 189, endnote 1).

2. The Russian federal law ‘On Protecting Children from Information Harmful to Their Health and Development’, forbidding the ‘propaganda of non-traditional sexual relationships’ among minors, was introduced in summer 2013, and has been circulated in US media discourse as the ‘Russian anti-gay law’ or ‘anti-homosexual propaganda law’.

3. Western media as well as many Russian analysts understand the contemporary Russian Orthodox Church and especially its leadership as in very close alliance with the government. Critical voices see the Church increasingly as strongly influencing official state policies; others argue that the Putin government uses the Church and its growing popularity to create greater approval of national policies and state actions

4. Pulitzer Center, 25 February 2015: https://pulitzercenter.org.

5. Derek Jarman was an English film director, stage designer, artist and author. Jarman, who was diagnosed with HIV in 1986, was a leading campaigner against the British ‘Clause 28’, which banned the ‘promotion of homosexuality’ (Peake, 1999: 418) in schools during the 1980s. He was an outspoken gay rights activist and, after being diagnosed, he also worked to raise awareness of HIV/AIDS in public until his death in 1994 (1).

6. See https://www.indiegogo.com/projects/the-world-according-to-homophobes#/.

7. They (them, their) is the preferred pronoun for many genderqueer and gender-fluid people in the English-speaking context.

8. The Commission on Security and Cooperation in Europe published in 2008 a briefing by Russian expert Edward Lucas to the Commission for the 110th Congress in 2011 that was titled ‘The New Cold War: Putin’s Russia and the Threat to the West’, indicating a new perspective on American–Russian relations.

9. For a discussion of the construction of US-American gay identities as white, non-Jewish or non-religious per se, and male, see also Halberstam (2016). Halberstam points out that in the wake of the shooting in a gay club in Orlando in summer 2016, media established a notion of gayness that is not only white and male, but also most threatened by violence and therefore in need of protection. This construction totally dismissed the fact that the audience of the club was mostly Latino. Moreover, it allowed for a focus on the non-white male perpetrator as Muslim.
